# Unusual Solitary Neurofibroma of Common Peroneal Nerve in a Child

**DOI:** 10.7759/cureus.33039

**Published:** 2022-12-28

**Authors:** Kashyap Kanani, Ratnakar Ambade, Suhas Landge, Aditya Pundkar, Rohan Chandanwale

**Affiliations:** 1 Department of Orthopaedics, Jawaharlal Nehru Medical College, Datta Meghe Institute of Medical Sciences, Wardha, IND

**Keywords:** children, peripheral nerve sheath tumour, tumour, common peroneal nerve, neurofibroma

## Abstract

Neurofibroma (NF) is a tumour of peripheral nerves, which would be seldom seen in the limbs, particularly in children's limbs. Soft, skin-coloured papules or small sub-mucosal nodules appear as these lesions. Neurofibroma is classified into three types: localized, diffuse, and plexiform. The vast majority of nerve injury is sporadic and localized, with an incredibly low risk of tumour formation. Neurofibromatosis can present as multiple skin lesions along with bone deformities in which a full investigation is critical where an undiscovered widespread illness may arise. This case study describes a neurofibroma on the common peroneal nerve of the left lower limb in a 6-year-old child who visited our hospital with chief complaints of pain and swelling around the left proximal leg.

## Introduction

The peripheral nerve serves as a conduit for axons from the peripheral nervous system's motor, sensory, and vegetative systems [1]. It carries data in both directions between these neurons and their peripheral effectors (sensory receptors, skeletal muscles, and viscera) [2]. The nerve motor content is represented by the afferences to the peripheral, whereas the nerve-sensitive content is represented by the efferences from the periphery, which are responsible for sending information to the central integrators [3]. The nerve sheath is a layer of myelin and connective tissue that surrounds and insulates nerve fibers. A nerve sheath form of cancer is that which develops within the cells of this protective coating [4]. Nerve tumour cells come in a variety of forms. Some are inheritable, even though the etiology is largely undefined. Peripheral nerve tumour cells affect nerves by expanding within them (intraneural tumours) or applying pressure against them (external nerve tumours) (extraneural tumours) [5]. The majority are benign, such as schwannoma, neurofibroma, and perineuroma. The majority of peripheral nervous system sheath tumours are intermittent, with no obvious cause. In other cases, those who are part of a genetic disorder consist of multiple nerve sheath tumours, such as NF1, NF2, or schwannomatosis [6]. Neurofibromas are the most prevalent tumours of the peripheral nerve sheath, impacting both men and women equally but without regard to race or ethnic background [7]. The age at diagnosis varies greatly; however, localized lesions are quite common among people aged 20 to 40 years. The dispersed and plexiform forms are more common among children, with the plexiform form happening only very rarely after the age of 5 [8]. Lesions around the axilla and groin are common in neurofibromatosis type 2 whereas internal tumours are deeper and can be around the eye, associated with the gastrointestinal (GI) tract, retroperitoneal, or in the mediastinum [7]. We report the case of a 6-year-old child with a solitary peripheral nerve sheath tumour of the lower limb arising from the common peroneal nerve.

## Case presentation

**Figure 1:**
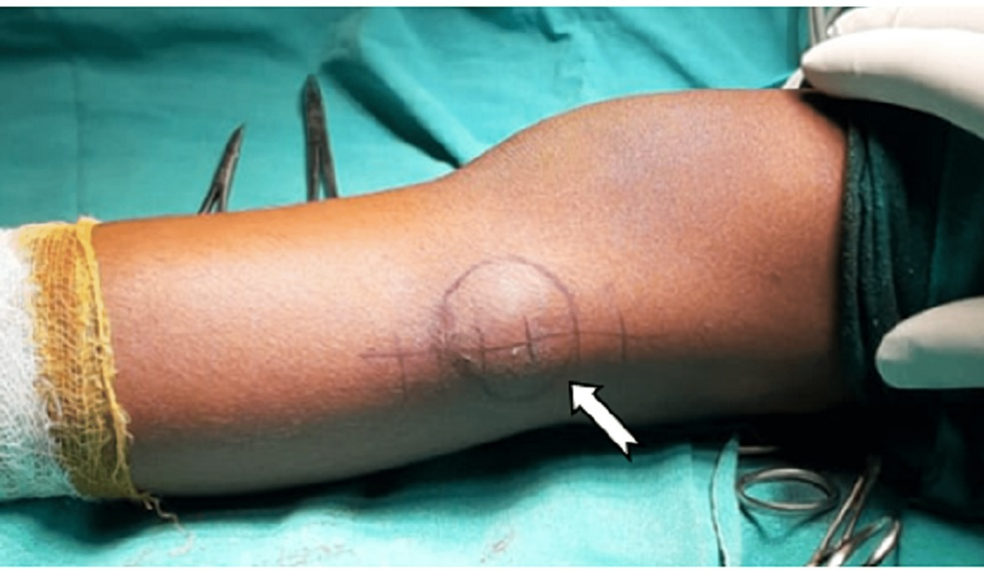
Clinical site of the tumour (lateral view of the left knee)

A 6-year-old boy presented to the Orthopaedics Outpatient Department with complaints of pain and swelling on the lateral aspect of the left knee just around the head of the fibula that had been present for three years. The parents reported early symptoms of painless swelling over the lateral aspect of the left knee, which gradually grew larger (Figure 1). For the last two months, the patient has been experiencing pain and discomfort as a consequence of the swelling. The parents gave no history of trauma or any other lesion over the body. There was no other significant family history.

**Figure 2:**
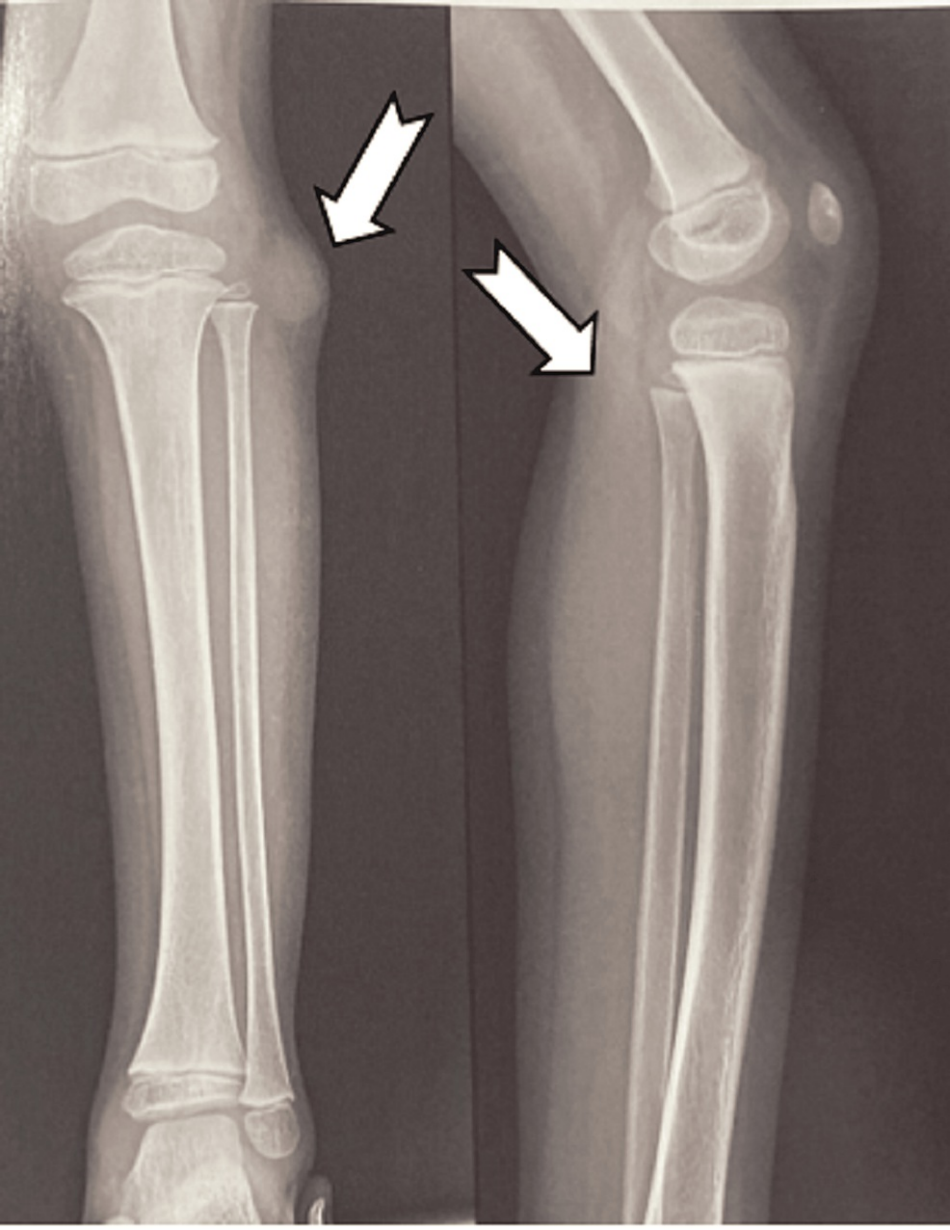
X-ray of left knee with leg anteroposterior and lateral view showing soft tissue swelling at the lateral aspect of the proximal leg near the head of the fibula

**Figure 3:**
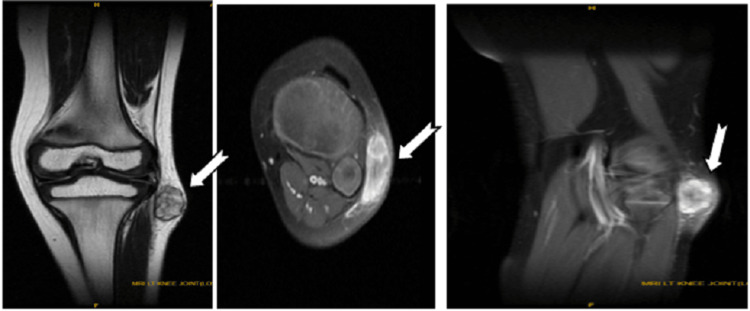
MRI with contrast of knee showing the tumour

The patient was further radiographically evaluated with an X-Ray of the left knee with leg (Figure 2) and MRI (Figure 3) and was tentatively diagnosed with peripheral nerve sheath tumour. Cytology from fine-needle aspiration revealed a benign nerve sheath tumour neurofibroma with cystic degeneration.

**Figure 4:**
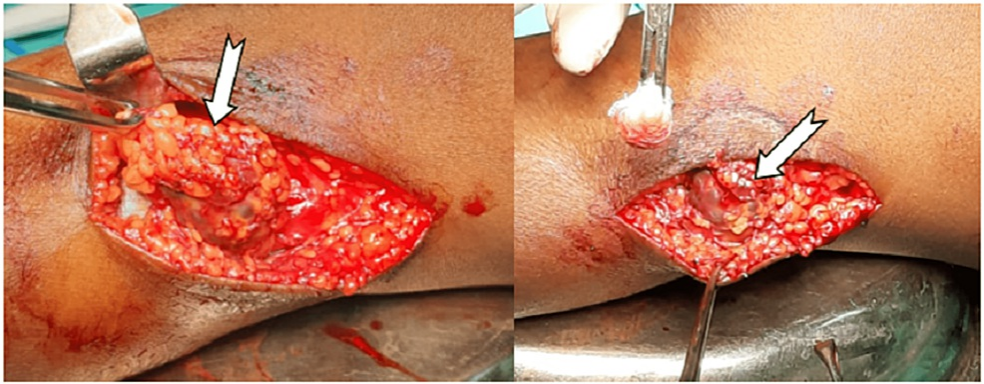
Location of the tumour arising from a common peroneal nerve

**Figure 5:**
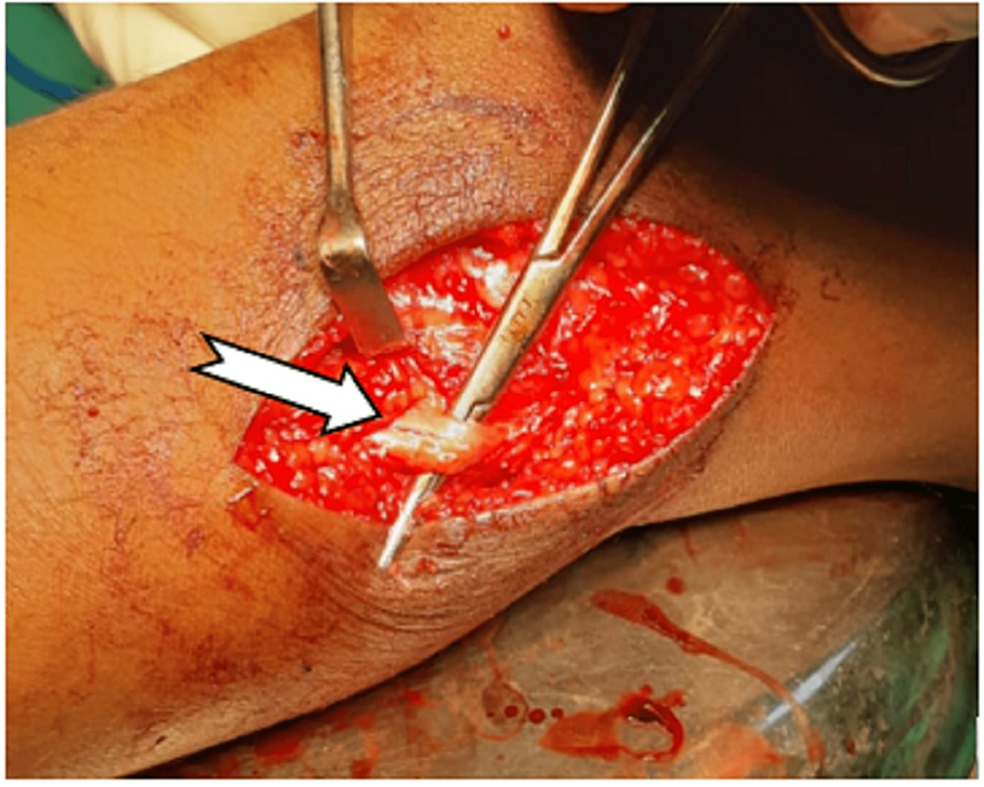
Location of common peroneal nerve where the tumor was attached

The patient was scheduled for tumour excision, and it was discovered intraoperatively that the tumour was originating from the common peroneal nerve sheath (Figures 4, 5).

**Figure 6:**
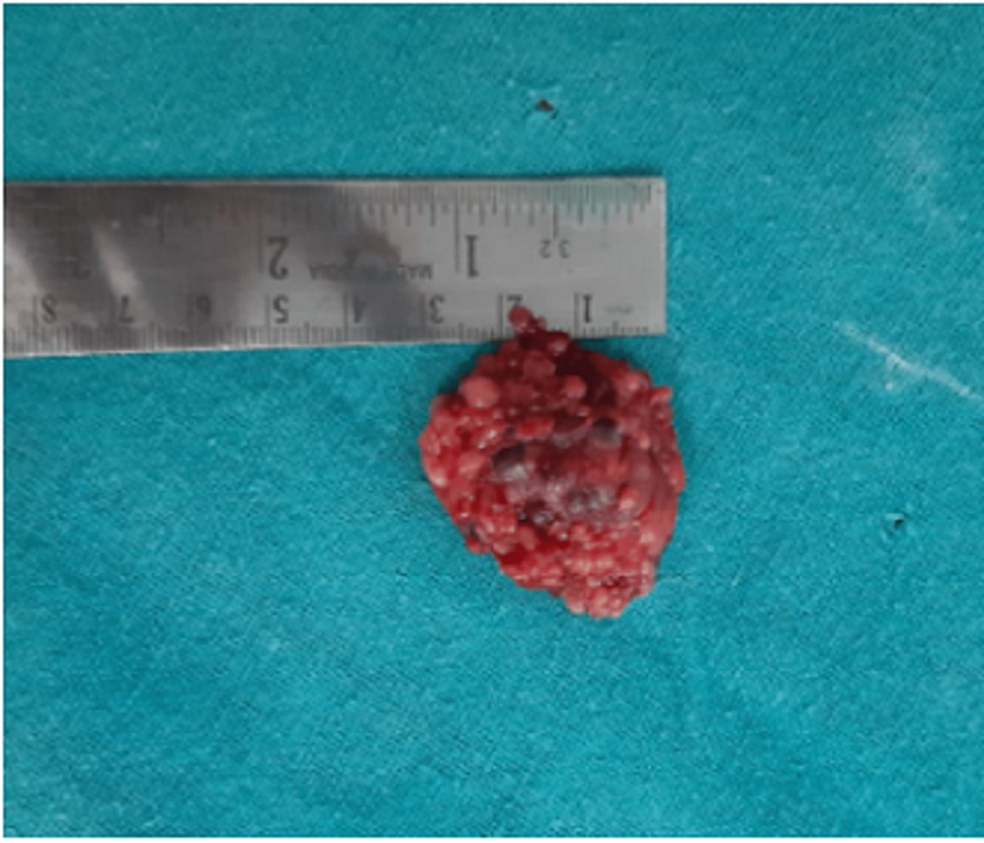
Excised tumour in total

A 4 cm x 3 cm tumour was resected and sent for histopathological examination, which revealed significant autolytic changes and histopathological features consistent with a benign nerve sheath tumour (neurofibroma) (Figure 6). There was no neurological or functional deficit as a result of the surgical procedure. The patient was started on full weight-bearing mobilization from post-operative day 1. The patient was followed up for six months and had no complaints of pain and no signs of recurrence.

## Discussion

Benign nerve layer tumours are divided into two kinds based on their morphology and histopathology: schwannoma (also recognized as neurilemoma or neurinoma) and neurofibroma. An innocuous peripheral nerve sheath neoplasm with classic distinguishing characteristics, such as the presence of neurons in the brain component made up of changed Schwann cells and a benign tumour fibrous element made up of fibroblasts. 90% of isolated neurofibromas are sporadic or occur as a portion of neurofibromatosis type 1, and 10% are inherited [9]. Deep granulomas are less prevalent than surface-level neurofibromas. Neurofibromas are noncancerous nerve sheath tumour cells with a tan-white, glittering cut surface. Their growth model is either well-defined intraneural or dispersed soft tissue incursion at extraneural sites [6]. They seem to be fairly common, especially at segmentation in dividing sites where they manifest as localized, polyp growths. Region-specific, disperse, and plexiform are the clinical and pathological subgroups based on architecture and design growth patterns [10].

Neurofibromas are benign, sluggish tumours. They originally come in the nerve and are completely composed of Schwann cells in a collagenous matrix. Those who typically exhibit firmness, delimitation, and encasing. Small neurofibromas are spheroidal in structure, although bigger tumour cells could be ovoid, sausage-shaped, or inconsistently lobulated [11]. A combination of peripheral nerve sheath tumour cells (PNSTs) is benign PNSTs that have attributes of much more than one type of noncancerous PNST, such as neurofibroma, schwannoma, and perineurium. One of the most popular types is sporadic configurations of Schwannoma/perineurial and neurofibroma/schwannoma, which are traditionally associated with neurofibromatosis (NF) type 1 or 2 or schwannomatosis [12]. Neurofibroma/perineurium combinations are unusual and are generally linked with NF1. The most common type is localized cutaneous neurofibroma, which occurs infrequently in the vast majority of cases. Localized neurofibromas can necessitate a significant nerve and cause fusiform growth of the nerve trunk (intraneural subtype) [13]. The peripheral nerves of the limbs, particularly the common peroneal nerve, are typically unaffected [14]. Neurofibromas are usually associated with neurofibromatosis type 1, with a prevalence of 1 in 3000 births, and also associated with neurofibromatosis type 2, with a prevalence of 1 in 33,000 births [7]. When compared to other body surfaces, solitary neurofibromas are known to favour the trunk and head. Some unusual sites of solitary neurofibroma reported are over the palms and plantar aspects of the foot and around the oral cavity [15]. Solitary neurofibroma of the thigh has been reported where the patient presented with painless swelling over the thigh which had no vascular involvement and was excised successfully [16]. Here we found a solitary neurofibroma of the common peroneal nerve near the head of the fibula in a 6-year-old child which was not associated with any neurological involvement of the nerve and was excised successfully. So this must be taken into account in the differential diagnosis of swelling all around the knee and pain or numbness or tingling of the leg and foot.

## Conclusions

Schwannoma, as well as neurofibromas, are the most commonly diagnosed peripheral sheath benign tumours. Findings of neurofibroma emerging from the common peroneal nerve, on the contrary side are exceedingly rare. We discovered a solitary benign neurofibroma of the common peroneal nerve, which was effectively excised with no neurological or functional deficits.
